# Error-Related Dynamics of Reaction Time and Frontal Midline Theta Activity in Attention Deficit Hyperactivity Disorder (ADHD) During a Subliminal Motor Priming Task

**DOI:** 10.3389/fnhum.2019.00381

**Published:** 2019-10-29

**Authors:** Marius Keute, Max-Philipp Stenner, Marie-Kristin Mueller, Tino Zaehle, Kerstin Krauel

**Affiliations:** ^1^Department of Neurology, Otto-von-Guericke-University, Magdeburg, Germany; ^2^Department of Child and Adolescent Psychiatry and Psychotherapy, Otto-von-Guericke-University, Magdeburg, Germany; ^3^Department of Behavioral Neurology, Leibniz Institute for Neurobiology, Magdeburg, Germany; ^4^Center for Behavioral Brain Sciences, Magdeburg, Germany

**Keywords:** ADHD, EEG, frontal midline theta, post error slowing, performance monitoring

## Abstract

Post-error slowing (PES) is an established performance monitoring readout. Several previous studies have found that PES is reduced in children and adolescents with attention-deficit hyperactivity disorder (ADHD). We analyzed reaction time data, along with electroencephalography (EEG) data, from a response priming experiment in children and adolescents with ADHD (*N* = 28) and typically developing (TD) controls (*N* = 15) between 10 and 17 years of age. We report dynamic reaction time changes before and after errors: whereas TD controls readjusted their response speed to their individual average speed after committing an error, this reaction time adjustment appeared to be delayed and decreased in ADHD patients. In the EEG, error trials were accompanied by increased frontal midline theta activity, which was attenuated in ADHD compared to TD. We conclude that PES has a different time course rather than being fully absent in ADHD and discuss relationships with our EEG findings and potential implications for performance monitoring in ADHD.

## Introduction

Performance monitoring is an essential feature of human behavior, enabling individuals to constantly evaluate whether their current behavior and its outcomes are still in line with personal goals or task demands, and to adjust behavior, if necessary. Performance monitoring has often been studied in reaction time experiments, where an extensively scrutinized phenomenon is referred to as post-error slowing (PES), i.e., the tendency to slow behavioral responses after a preceding erroneous behavioral response. Even though the precise mechanism and function of PES are controversial, there is wide agreement that PES reflects performance monitoring processes, in that it serves to inhibit premature post-error responses and to provide the individual with time for attentional reallocation and performance improvement (Danielmeier and Ullsperger, 2011). An alternative (but not necessarily conflicting) account of PES states that errors require attentional reorientation because they are infrequent events, and that this reorientation causes reaction time costs, leading to PES (Notebaert et al., 2009).

It has been found that individuals tend to increase their response speed before an error (Dudschig and Jentzsch, 2009). This finding suggests that there is a dynamic sequence of response speeding and slowing around errors rather than an isolated PES. These peri-error changes in response speed can be conceptualized as a shift in speed-accuracy trade-off: as long as no error occurs, responses are speeded at the expense of increasing error likelihood. Conversely, occurrence of an error signals that a readjustment to slower but more accurate responses is necessary.

Errors are accompanied by a transient increase in theta band (4–8 Hz) brain oscillations as measured by electroencephalography (EEG) at fronto-central sites (Cavanagh and Frank, 2014). Often referred to as frontal midline theta (FMΘ), it serves as a well-established neurophysiological marker of increased cognitive control and performance monitoring. It is not restricted to error commission, but can also be found during response conflicts and other situations requiring increased control. The anterior cingulate cortex (ACC) and medial prefrontal cortex have been identified as its principal neural sources (Asada et al., 1999; Cohen, 2011; Cavanagh and Shackman, 2015). Error-related FMΘ has been linked to PES, in that both subserve performance recovery following errors (Valadez and Simons, 2018).

Attention deficit hyperactivity disorder (ADHD) is one of the most prevalent psychiatric conditions in children and adolescents, characterized by symptoms of inattention, impulsivity and deficient and volatile executive functioning, including deficits in performance monitoring (Liotti et al., 2005; McLoughlin et al., 2009). In a recent meta-analysis of PES in ADHD patients (Balogh and Czobor, 2016), the majority of included studies found PES to be decreased in ADHD compared to typically developing (TD) controls. Some studies, however, found intact PES in ADHD (Wiersema et al., 2005). The presence and magnitude of PES in healthy individuals and its alterations in ADHD patients depend on parameters of the task, such as task difficulty, error type (omission vs. commission), and inter-stimulus interval (Balogh and Czobor, 2016), as well as intraindividual factors such as error awareness and response-stimulus interval (Danielmeier and Ullsperger, 2011). Whether or not error awareness is necessary for PES to occur is controversial (Nieuwenhuis et al., 2001; Endrass et al., 2005). In most studies, error awareness is ensured through feedback signals (Notebaert et al., 2009). Deficient feedback processing (Groen et al., 2008) and error awareness (O’Connell et al., 2009) are potentially relevant factors for PES deficits in ADHD. Error monitoring and PES are modulated by dopamine neurotransmission (Krämer et al., 2007; Wardle et al., 2012), which is also involved in ADHD pathophysiology (Li et al., 2006; Genro et al., 2010) and therefore is a potential neural mechanism underlying PES deficits in ADHD. Moreover, several EEG studies found the error-related negativity/positivity complex (ERN/Pe), which is largely identical to the evoked (i.e., phase-locked) proportion of FMΘ (Luu et al., 2004), to be reduced in amplitude in ADHD (Liotti et al., 2005; Wiersema et al., 2005; Groom et al., 2010; Geburek et al., 2013), which suggests that functional and structural impairments in the mid-frontal cortex (Tian et al., 2006; Bonath et al., 2018) contribute to PES deficits in ADHD.

As described above, PES is not an isolated post-error phenomenon, but rather occurs as part of a dynamic sequence of RT changes around errors. This raises the question of whether PES deficits in ADHD reflect alterations of the entire peri-error RT dynamics, or are specific to the pre-error or post-error period. With respect to the pre-error period, it is conceivable that increased intraindividual RT variability (Kofler et al., 2013) and an impaired ability to regulate speed-accuracy tradeoffs (Mulder et al., 2010) preclude the systematic build-up of pre-error speeding in ADHD patients, so that errors could occur more “surprisingly” compared to TD controls, without a preceding period of speeded responses. Conversely, in the post-error period, deficient feedback processing and error awareness (Groen et al., 2008; O’Connell et al., 2009) and a general deficit in action regulation (Shiels and Hawk, 2010; Barkley, 2011) may hinder ADHD patients from slowing RT.

We conducted secondary analyses of error-related behavioral and EEG data from a motor priming task. The primary study (Keute et al., 2018) has been designed to study automatic motor inhibition in ADHD.

Furthermore, we explored differences between ADHD subtypes with respect to peri-error RT changes, i.e., between patients diagnosed with the predominantly inattentive subtype and patients with pronounced hyperactive-impulsive symptomatology (predominantly hyperactive-impulsive and combined subtypes). Even though we have no specific hypothesis about ADHD subtypes, we want to explore a potential contribution of this symptomatology to error-related RT changes, since at least one study found PES deficits to be limited to the inattentive subtype (Shiels et al., 2012), whereas most other studies do not consider ADHD subtypes or report no differences between them (Balogh and Czobor, 2016). We studied FMΘ in error and peri-error periods as a potential neurophysiological correlate of peri-error performance dynamics, expecting decreased error-locked FMΘ power in ADHD patients, consistent with the above findings of reduced ERN/Pe complex amplitudes.

## Materials and Methods

### Experimental Procedure

We carried out a combined behavioral and EEG experiment. Prior to the experiment, written informed consent was obtained from the participants’ parents, and written informed assent was obtained from the participants. They received a €10 voucher for a local shopping center and were reimbursed for any traveling costs. The study was carried out in accordance with the Declaration of Helsinki and has been approved by the ethics committee of the medical faculty at the Otto-von-Guericke University Magdeburg.

Participants were recruited from an established participant pool of the Department of Child and Adolescent Psychiatry and Psychotherapy at the university hospital in Magdeburg. Prior to admission to this pool, each individual (patients as well as TD) underwent a clinical diagnostic routine, encompassing separate clinical interview with the parent and the child/adolescent (Schedule for Affective Disorders and Schizophrenia for School-Age Children—Present and Lifetime Version, K-SADS-PL, Kaufman et al., 1997; German adaptation, Delmo et al., 2000), clinical symptom checklists (Child Behavior Checklist, CBCL, Achenbach, 1991a; Youth Self Report, YSR, Achenbach, 1991b), a test for sustained visual attention (d2-R, Brickenkamp et al., 2010), and the culture-fair intelligence test (CFT 20-R, Weiß, 2008; see Table 1). According to DSM-IV, individuals were diagnosed with ADHD when they fulfilled at least six out of nine criteria for inattentiveness and/or at least six out of nine criteria for hyperactivity/impulsivity for more than 6 months, experienced first symptoms before the age of 7, and were significantly impaired in at least two settings.

**Table 1:** Sample description.

	ADHD	TD	*t* (p)
Number	32	18
Age (years)	13.7	13.6	−0.34 (0.73)
Sex ratio (M/F)	28/4	15/3
Intelligence	101.3 (12.3)	110.4 (11.9)	2.56 (0.015)
Attentional performance (*T*-values)	52.8 (8.5)	62.3 (10.7)	3.43 (0.003)
YSR (*T*-values)
Attentional Problems	62.9 (8.6)	53.5 (3.6)	−4.87 (>0.001)
Dissocial Behavior	54.5 (5.2)	53.8 (5.3)	−0.37 (0.717)
Aggressive Behavior	56.6 (7.7)	53.3 (4.9)	−1.65 (0.107)
CBCL (*T*-values)
Attentional Problems	69.0 (8.6)	53.7 (4.3)	−8.35 (>0.001)
Dissocial Behavior	58.5 (6.6)	52.7 (4.6)	−3.65 (0.001)
Aggressive Behavior	61.4 (7.6)	53.6 (5.0)	−4.42 (>0.001)
Clinical diagnoses: ADHD	18 combined/		
	1 hyperactive		
	13 inattentive		
Oppositional defiant disorder	5		
Stimulant medication	8		

*YSR, Youth Self ReportCBCL, Child Behavior Checklist; Attentional performance, d2/d2-R*.

Participants (ADHD as well as TD) were excluded in case of any known past or present neurological disorder or substance abuse. Control participants were excluded if there was evidence of any previous or current psychiatric disorder. Overall, 32 children with ADHD (four females) and 18 TD controls (three females) participated. Participants were 10–17 years of age (M 13.7, SD 1.9). Both groups did not differ in mean age (see Table 1). Thirteen ADHD patients were diagnosed with the predominantly inattentive subtype (ADHD-I, number of inattentive symptoms ≥6, number of hyperactive/impulsive symptoms <6), 18 patients with the combined subtype (ADHD-C, number of inattentive symptoms ≥6, number of hyperactive/impulsive symptoms ≥6), and one patient with the predominantly hyperactive-impulsive subtype (ADHD-H, number of inattentive symptoms <6, number of hyperactive/impulsive symptoms ≥6). Combined and predominantly hyperactive-impulsive subtypes were aggregated in one group (ADHD-H/C). Six ADHD-C patients received a current comorbid diagnosis (five patients with oppositional defiant disorder, one patient with enuresis). Six patients in the ADHD-H/C group and two patients in the ADHD-I group were under psychostimulant treatment (methylphenidate or dexamphetamine), which they withheld for at least 24 h prior to the experiment. TD participants were unmedicated except for one boy who received a topic antibiotic for acne. All participants had normal or corrected-to-normal vision.

**Figure 1:**
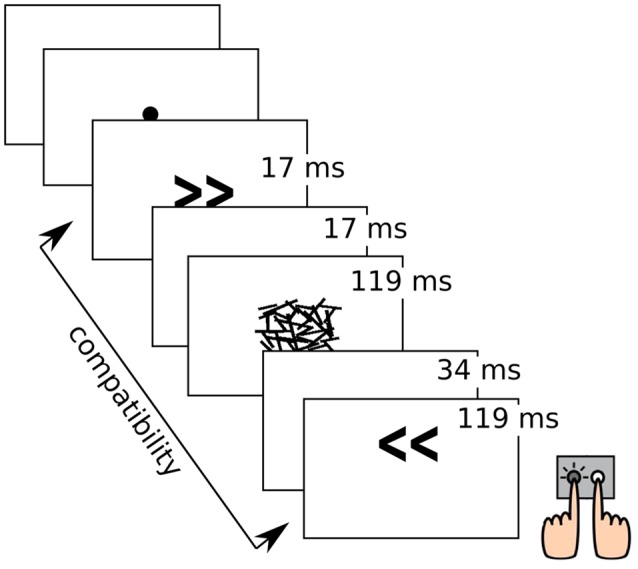
Illustration of the experimental task with stimulus durations. Object sizes are not true to scale.

Participants performed a subliminal motor priming task in which left- and right-pointing arrows (target stimuli) were presented on the screen, each of which was preceded by another arrow serving as priming stimulus. Priming arrows could point to the same (compatible) or opposite (incompatible) direction compared to the target arrow. Priming arrows were rendered subliminal by a random pattern mask. The task instruction was to indicate the direction of the target arrow by striking a key with the respective index finger. After incorrect or missed responses, a red “x” appeared on the screen as feedback. Participants were not informed about the presence of priming stimuli in advance, and in a subsequent prime recognition task following debriefing, only few participants were unable to identify directions of the masked primes with above-chance accuracy. The task comprised 288 trials in three blocks, with left-/right-pointing targets and compatible/incompatible primes in equal proportions and randomized order, respectively. Trials with wrong or missed responses were repeated at the end of each block, until 288 trials with correct responses were collected. This experimental task is known to elicit the so-called Negative Compatibility Effect (NCE), i.e., performance improvements through incompatible priming and performance costs through compatible priming, whereas intuitively, the opposite effect would be expected. This reversal of the (positive) priming effect is modulated by the stimulus onset asynchrony (SOA) between the prime-mask-stimulus and the target stimulus and explained through an automatic and unconscious motor inhibitory process (Eimer and Schlaghecken, 1998; Panis and Schmidt, 2016). The task design is illustrated in Figure 1.

We found that the NCE was neither absent nor significantly decreased in ADHD patients compared to TD controls, which indicates that the low-level motor inhibition process underlying the NCE is intact in ADHD. Behavioral and lateralized readiness potential (LRP) results have been described in more detail elsewhere (Keute et al., 2018).

### RT Analyses

For the analysis of error-related RT changes, we only considered commission errors that were “isolated,” i.e., preceded and followed by at least three correct trials (referred to as ER ± 1–3), respectively, in order to avoid potential confounds through overlapping effects of multiple subsequent errors on RT. Correct trials with at least three more correct trials distance to errors in either direction are referred to as “error-free” trials in the following. Only participants with five or more isolated errors were included in the analysis (resulting in a sample of 14 TD controls and 26 ADHD patients entering the final analyses). Trials that were neither error-free nor isolated errors nor ER ± 1–3 trials were excluded from further analyses.

For all correct responses, we standardized RTs by subtracting participant-wise mean RT calculated across error-free trials, and dividing by its standard deviation (analog to the computation of *z*-scores). We analyzed raw and standardized reaction times using linear mixed-effects regression models. To account for correlations between repeated measurements (trials) from the same individual, the models incorporated random intercepts per participant (Bates et al., 2015). Error rates were compared between groups by constructing a logistic linear mixed-effects regression model of trial-wise response-correctness with random intercepts per participant (Jaeger, 2008). We chose to analyze trial-wise RT data instead of participant-wise average values to account for varying number of trials between participants (see below). Statistical significance of effects in mixed-effects models was assessed by model comparisons using likelihood-ratio ratio tests. Next to effect sizes for significant effects, we report the test statistics (log-likelihood ratio/χ^2^) and *p*-values (Bates et al., 2015). For *post hoc* comparisons, we refitted and compared the models of interest with a subset of the data (e.g., with data from ADHD patients only).

Since linear mixed-effects regression is a relatively unconventional method, we repeated the most important analysis steps using mixed analysis of variance (ANOVA) over subject-wise mean values, which is more common, in order to demonstrate the equivalence of both analysis approaches.

### EEG Measurement and Analysis

EEG was recorded from eight scalp Ag/AgCl electrodes (F3, Fz, F4, C3, Cz, C4, O1 and O2 according to the international 10-20-System), mounted to an electrode cap (Easycap, Herrsching, Germany), with a right earlobe reference and a 0.05–70 Hz online bandpass filter at a sampling rate of 500 Hz, using a SynAmps amplifier (Compumedics Neuroscan, Victoria, Australia). Vertical and horizontal EOG were recorded from two electrodes each, using bipolar channels. Impedances were kept below 2 kΩ. Electrode selection was held similar to Eimer and Schlaghecken (1998).

EEG data were analyzed using FieldTrip (Oostenveld et al., 2011). Data were notch (48.5–51.5 Hz), and bandpass (0.3–40 Hz, 4th order Butterworth filter) filtered. A joint decorrelation procedure (de Cheveigné and Parra, 2014) was used to remove ocular artifacts. For this purpose, the position of eyeblinks was determined by peak detection in the vertical EOG channel, verified by visual inspection and fed into the algorithm to allow targeted decorrelation of EEG data and eyeblinks. Data were segmented into single trials (from −2 to +2 s relative to responses), and remaining artifact-laden trials were excluded based on a ± 80 μV threshold criterion.

Time-frequency analysis was carried out using Fast Fourier Transformation (FFT) over Hann-tapered time windows (0.5 s), moving from −1.5 to +1.5 s relative to responses in steps of 50 ms, at frequencies ranging from 2 to 36 Hz in steps of 2 Hz. Resulting spectral power values were log-transformed (10*log_10_), so that differences between log-power values have decibel (dB) units. Time-frequency data were baseline corrected to a trial-wise pre-prime baseline (−0.75 to −0.25 s relative to primes, note that this baseline included data ranging from −1 to 0 s relative to the prime, given the window length of 0.5 s). Data segments (trials) were sorted according to their position relative to errors (ER ± 1–3, error-free). A mean number of 13.8 isolated error trials (and ER ± 1–3 trials, respectively) were available per participant (SD 5.4, range 5–25).

Following initial validation by visual inspection, FMΘ was computed by averaging time-frequency data over theta frequencies (4–8 Hz) and electrodes Fz and Cz. To compare FMΘ between groups over the ER ± 1–3 range, we computed time-averaged theta power across a ±0.4 s time window relative to responses. For statistical inference, linear mixed-effects models were used, analog to the analysis of RT (see above). Models were fitted on participant-wise averaged theta power in ER ± 1–3 trials, compared to theta power in error-free trials.

All datasets and analysis scripts are available from the corresponding author on reasonable request.

### Power Analysis

This study was based on secondary analyses, therefore the sample size was given in advance. Nonetheless, we conducted a power analysis in the interest of transparency. Balogh and Czobor (2016) report a mean effect size (Cohen’s d) of 0.42 for PES group differences (ADHD vs. TD). When considering a *t*-test comparing PES between groups, using our sample size (26 ADHD, 14 TD) and a significance level of *p* = 0.05, statistical power at an effect size of 0.42 was at 23.5%. A normative 80% power level was achieved only for effect sizes of 0.95 and above.

## Results

### Behavioral Results

#### Overall Behavioral Performance

**Figure 2:**
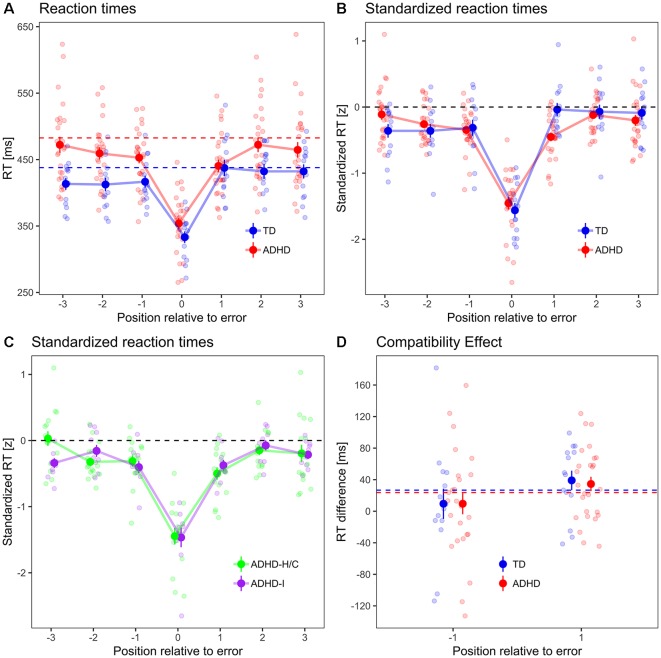
**(A)** Peri-error RTs, **(B)** standardized RTs, **(C)** standardized RTs in ADHD subtypes. Dots with error bars indicate mean ± standard errors of RT in error trials and in three preceding and subsequent trials, respectively, for typically developing (TD) controls (blue) and ADHD patients (red). Dashed lines indicate the mean error-free RT for each group (which is, by definition, zero for standardized RT). Small dots in the background indicate participant-wise mean values. **(D)** Compatibility effect in ER−1 and ER+1 trials. Dashed lines indicate compatibility effect from error-free trials in both groups.

Mean percentage of error responses was 9.7 (SD 5.2) in ADHD patients and 6.8 (SD 4.1) in TD controls. The difference in accuracy between groups missed statistical significance (*χ*^2^ = 3.3, *p* = 0.068). Error-free RT was lower in TD controls (427 ms, SD 31 ms) than in ADHD patients (459 ms, SD 41 ms; *t*_(34)_ = 2.8, *p* = 0.008, Figure 2A). In error trials, standardized RT was 1.39 SD below error-free mean RT (*χ*^2^ = 83.8, *p* < 0.001) and did not significantly differ between ADHD and TD (*χ*^2^ = 0.6, *p* = 0.429). Error percentage in trials following errors was 11.1 (SD 8.5). It was higher in TD controls compared to ADHD patients (*t*_(34)_ = 2.7, *p* = 0.010).

#### Peri-Error Behavioral Performance

Analyses of peri-error performances (three trials preceding and following errors, respectively) have been carried out using standardized RT (see “Materials and Methods” section) in order to facilitate comparisons of intra-individual RT changes. Standardized RT (Figure 2B) in pre-error trials was, across groups, 0.26 SD below zero, i.e., below individual error-free RT (*χ*^2^ = 36.2, *p* < 0.001), but did not differ between TD and ADHD (*χ*^2^ = 0, *p* = 0.895). It decreased by 0.1 SD/trial between the ER−1, ER−2, and ER−3 trial (*χ*^2^ = 6.7, *p* = 0.009). The trial×group interaction missed statistical significance (*χ*^2^ = 3.2, *p* = 0.075). This means that errors were, across groups, preceded by a period of increasing response speed across at least three trials.

Across groups, post-error standardized RT was 0.16 standard deviations below zero (*χ*^2^ = 14.4, *p* < 0.001). It was 0.16 SD lower in ADHD patients compared to TD controls (*χ*^2^ = 4.2, *p* = 0.040), and increased by 0.08 SD/trial between the ER+1, ER+2 and ER+3 trial (*χ*^2^ = 6.5, *p* = 0.011). There was a group×trial interaction (*χ*^2^ = 6.9, *p* = 0.008).

*Post hoc* tests revealed that standardized RT in ER+1 trials was 0.44 SD below zero in ADHD patients (*χ*^2^ = 71.8, *p* < 0.001), but was not significantly different from zero in TD controls (*χ*^2^ = 0, *p* = 0.978). Comparing ER−1 and ER+1 trials group-wise, standardized RTs were not significantly different between trials in ADHD patients (*χ*^2^ = 0.5, *p* = 0.464), whereas in TD controls, standardized RT increased by 0.13 SD from ER−1 to ER+1 trials (*χ*^2^ = 5.2, *p* = 0.023), i.e., there was a pre-to-PES in TD controls but not in ADHD patients. However, ADHD patients slowed responses between ER+1 and ER+2 trials by 0.42 SD (*χ*^2^ = 25.1, *p* < 0.001), and their standardized RT in ER+2 trials was not significantly different from zero anymore (*χ*^2^ = 0.6, *p* = 0.422).

We repeated the main analyses using mixed ANOVA. Comparing subject-wise mean RT in ER−1 and ER+1 trials in a group (TD, ADHD) × trial (ER−1, ER+1) mixed ANOVA, we found no main effects of group and trial position (*F*_(1,38)_ < 2.5, *p* > 0.12), but a significant interaction (*F*_(1,38)_ = 5.5, *p* = 0.024). The same analysis for standardized RT yielded a main effect of group (*F*_(1,38)_ = 6.7, *p* = 0.012), no significant main effect of trial position (*F*_(1,38)_ = 0.1, *p* = 0.735), and a significant interaction effect (*F*_(1,38)_ = 5.1, *p* = 0.030). The *post hoc* contrast within the ADHD group showing that there is a PES between the ER+1 and ER+2 trial can also be replicated using a paired *t*-test (*t*_(25)_ = 3.8, *p* < 0.001).

#### ADHD Subtype Comparison

The peri-error RT pattern was not fundamentally different between ADHD subtypes (Figure 2C): there was no main effect of ADHD subtype on standardized pre-error RT (*χ*^2^ = 1.2, *p* = 0.277), nor an interaction between subtype and pre-error trial position (*χ*^2^ = 0.1, *p* = 0.767). Likewise, in post-error trials, there was neither a significant main effect of subtype on standardized RT (*χ*^2^ = 1.9, *p* = 0.168), nor an interaction between subtype and trial position (*χ*^2^ = 0.3, *p* = 0.601).

#### Compatibility Effect

The compatibility effect (Figure 2D), i.e., the RT difference between trials with compatible and incompatible priming, differed between pre- and post-error trials by 27 ms (higher in post-error trials, *χ*^2^ = 4.4, *p* = 0.037), but not between groups (*χ*^2^ = 0, *p* = 0.870), nor did trial position interact with group (*χ*^2^ = 0, *p* = 0.861).

### EEG Results

**Figure 3:**
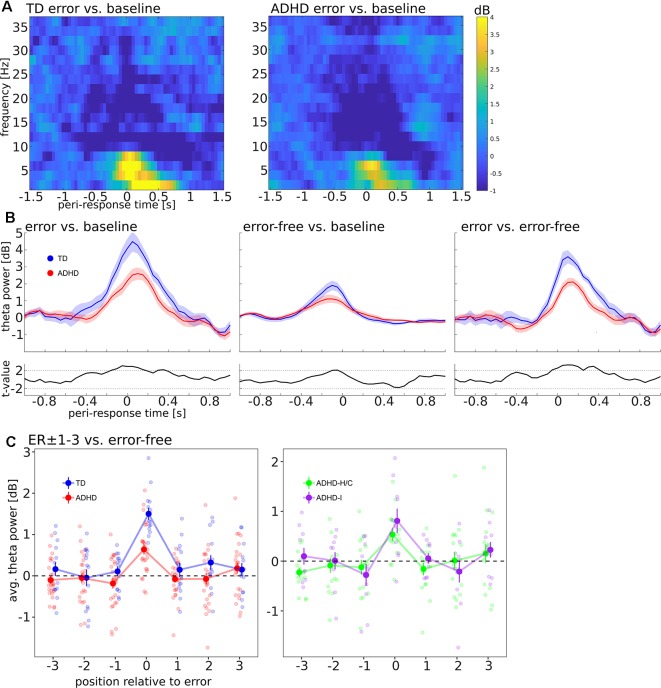
**(A)** Baseline-corrected time-frequency representation, locked to error responses. Panels show log-power for frequencies from 2 to 36 Hz in steps of 2 Hz, averaged over electrodes Fz and Cz, compared to a pre-cue baseline (−750 to −250 ms). Left panel: TD, Right panel: ADHD. **(B)** Mean ± SEM of change in the theta band, averaged over frequencies (4–8 Hz) and electrodes (Fz, Cz). Left and middle panel show error and error-free trials, respectively, compared to pre-cue baseline, the right panel shows error compared to error-free trials. Blue Curves: TD, Red curves: ADHD, black curves below: *t*-values comparing TD and ADHD; dashed black lines indicate *t* = 2.02, the (uncorrected) threshold for two-tailed statistical significance at *df* = 38. **(C)** Time-averaged theta power (−400 to +400 ms relative to responses) in peri-error trials in TD vs. ADHD (left) and compared between ADHD subtypes (right).

Visual inspection of the response-locked time-frequency representations for error trials (Figure 3A) revealed a transient theta band (4–8 Hz) power increase over ca. ±400 ms relative to the error response across groups.

Figure 3B shows FMΘ dynamics in error and error-free trials. It can be seen that numerically, the response-locked theta power increase was attenuated in ADHD patients compared to TD controls in error as well as error-free trials. Accordingly, *t*-values (Figure 3B, small panels) exceeded the threshold for (uncorrected) statistical significance in these trials around the time of the theta peaks, and time-averaged FMΘ in ADHD patients was significantly smaller in error-trials (error vs. baseline contrast: *t*_(38)_ = −2.83, *p* = 0.008; error vs. error-free contrast: *t*_(38)_ = −4.35, *p* < 0.001). Across groups, FMΘ was significantly different from zero in error-trials (*t*_(39)_ = 8.16, *p* < 0.001). In error-free trials (baseline contrast), FMΘ was significantly different from zero as well (*t*_(39)_ = 4.28, *p* < 0.001), but the group difference did not approach statistical significance (*t*_(38)_ = −0.05, *p* = 0.962).

Considering the ER ± 1–3 region (error-free contrast, Figure 3C, left panel), we found that the intercept of time-averaged theta power in pre-error trials (ER−1–3) was not significantly different from zero (*χ*^2^ = 0.59, *p* = 0.441), nor were there main effects of group and trial position nor an interaction between group and trial position (all *χ*^2^ < 2.3, all *p* > 0.13). Likewise, in post-error trials (ER+1–3), neither the intercept of time-averaged theta power (*χ*^2^ = 1.51, *p* = 0.219), nor the main effects of group and trial position, nor the interaction between group and trial position approached statistical significance (all *χ*^2^ < 2.3, all *p* > 0.13).

Comparisons of FMΘ between ADHD subtypes (Figure 3C, right panel) did not reveal any significant subtype differences: FMΘ in error trials did not differ significantly between ADHD-H/C and ADHD-I (*t*_(15)_ = 0.99, *p* = 0.335). There was neither a subtype main effect nor an interaction between trial position and subtype, neither in the pre-error nor post-error period (all *χ*^2^ < 2.6, all *p* > 0.1). On the group level, we found no significant correlations between error-locked FMΘ and standardized RT in ER+1 trials (all participants: *r* = 0.21, *p* = 0.187; within TD group: *r* = −0.15, *p* = 0.606; within ADHD group: *r* = −0.09, *p* = 0.675).

## Discussion

We have presented an analysis of error-related reaction time and FMΘ dynamics in a response priming experiment involving ADHD patients and TD controls. We found that both groups alike speeded their responses prior to and during errors.

Crucially, ADHD patients and TD controls showed different patterns of post-error adjustment: TD controls showed pre-to-PES, readjusting response speed to their individual error-free mean RT. In ADHD patients, the pre-error fast-response period “survived” the error, in that responses remained speeded up in ER+1 trials and were slowed only in ER+2 trials. We conclude that post-error RT adjustments are delayed and decreased rather than being fully absent in ADHD. In the EEG data analysis, we found increased FMΘ responses in error trials that were attenuated in ADHD compared to TD. Compared to error-free trials, FMΘ was increased during errors but not in peri-error trials.

FMΘ has not been studied extensively in ADHD so far. One study investigated evoked (i.e., phase-locked) error-related FMΘ and found it to be attenuated in ADHD patients (Groom et al., 2010), and one study that decreased connectivity between FMΘ and posterior alpha oscillations in ADHD (Mazaheri et al., 2014). Both findings are consistent with our finding of attenuated total (i.e., phase-independent) error-related FMΘ power. Decreased FMΘ in ADHD is in line with previous findings of functional and structural deficits in the ACC and the medial prefrontal cortex, two brain regions involved in performance monitoring and the generation of FMΘ (Hauser et al., 2014; Holroyd and Umemoto, 2016; Bonath et al., 2018).

With respect to the RT findings, the fact that responses were speeded prior to and during errors across groups indicates that RT dynamics were likely shaped by a speed-accuracy trade-off (Dudschig and Jentzsch, 2009), as described in the “Introduction” section, that is, errors can be thought of as premature, hasty responses (Scheffers and Coles, 2000), rather than resulting from decision uncertainty and subsequent guessing, which would result in slowed error responses, as predicted by the so-called deadline model (Ruthruff, 1996; Mohamed et al., 2016).

Our finding of delayed and decreased post-error RT adjustments in ADHD patients allows for different interpretations. It might be due to a general deficit in executive control and behavioral adaptation (Willcutt et al., 2005; van Meel et al., 2007). It might as well be accounted for by a deficit in feedback processing and error-awareness (van Meel et al., 2005; Groen et al., 2008; O’Connell et al., 2009). However, the latter account does not appear to be supported by the EEG findings, in that FMΘ was increased during errors, compared to error-free trials, in both groups, which suggests that, in principle, errors were noticed and an initial activation of brain regions involved in performance monitoring occurred. On the other hand, it is not apparent from the FMΘ data that the same performance monitoring processes as in TD controls are simply “postponed” by one trial in ADHD, in that no group differences in FMΘ were found during post-error trials. An alternative account could be that the neuronal signal for behavioral adjustment that is reflected by FMΘ occurs around the same time across groups (i.e., during error commission), but ADHD patients require more time to implement the behavioral adjustment (reflected by PES), so that it occurs only two trials later instead of one. This account would be compatible with our behavioral and FMΘ findings, and it appears to be in line with previous findings of decreased processing speed in ADHD (Calhoun and Mayes, 2005; Shanahan et al., 2006;Goth-Owens et al., 2010).

Peri-error RT changes were not significantly different between ADHD subtypes, so we can conclude that the mechanisms of error-related RT changes are similar between ADHD subtypes.

These patterns of peri-error RT variability were independent of the experimental manipulation, i.e., the NCE induced through compatible and incompatible response priming. Across groups, there was a pre-to-post increase in the magnitude of the NCE. Even though it is beyond the scope of this report, this finding is in need of explanation, since it seems to contradict findings from experiments involving Eriksen Flanker tasks, where a post-error reduction in interference, i.e., a decreased compatibility effect, was found (Van der Borght et al., 2014). A possible explanation lies in the different role of response inhibition involved in the Eriksen Flanker task and the NCE: whereas in the Eriksen Flanker task, response inhibition is needed to suppress the compatibility effect induced by interfering irrelevant stimuli (flankers), with the NCE, response inhibition is causing the interference and compatibility effect in the first place, therefore it is not surprising that peri-error modulations of compatibility effects are different in both tasks. More detailed discussions of response inhibition and interference in the NCE have been published elsewhere (Schlaghecken et al., 2007; Sumner, 2007; Keute et al., 2018).

A potential limitation of this report is that it is based on secondary analyses, i.e., all analyses were conceived after data collection was complete. Therefore, the response priming paradigm was not initially designed to study error processing, and the resulting number of errors available for analysis per participant was relatively low. Even though previous studies have shown (for EEG data) that 12–14 error trials are, in principle, sufficient to yield reliable results, given a sample size of 32 individuals or more (Pontifex et al., 2010; Boudewyn et al., 2018), a higher amount of available data would strengthen our findings. Moreover, the EEG was recorded from only eight electrodes. While we chose this setup to reduce the distress coming with electrode preparation, especially for the ADHD patients, it poses a limitation inasmuch as a more dense EEG setup would have allowed for more sophisticated artifact correction and data analyses. Furthermore, more errors occurred in trials with compatible compared to incompatible priming. Even though we have shown that group differences in the peri-error RT dynamic are independent from prime compatibility, the low number of errors did not allow us to fully account for compatibility effects. A higher number of available error and peri-error trial per participant would also have allowed for trial-level analyses to better trace the functional relationship between RT and FMΘ dynamics, and would have strengthened our conclusions in general. On the group level, we did not find any correlations between error-locked FMΘ and post-error RT adjustments, neither across all participants nor in the ADHD nor TD subgroup.

Our sample size was limited, and the power analysis has shown that it was underpowered for typical effect sizes reported in the literature. Even though our conclusions are based on statistically significant results, a higher sample size would have been desirable. This is particularly true for our ADHD subtype comparison, which was based on an even smaller subset of the sample (ADHD patients). Finally, we did not systematically vary relevant experimental parameters such as task difficulty or presence and salience of error feedback. Given these limitations, we would like to stress the preliminary nature of our findings. We explicitly recommend independent replications of our findings in other experimental paradigms before strong conclusions can be drawn.

## Conclusion

In sum, we have found that deficient PES in ADHD actually reflects a lack of RT normalization in ER+1 trials, and that a partial normalization can be found between ER+1 and ER+2 trials. Error-related FMΘ dynamics are attenuated in ADHD, but this attenuation is selective for error trials and does not closely parallel the peri-error dynamics of RT.

## Data Availability Statement

The datasets generated for this study are available on request to the corresponding author.

## Ethics Statement

The studies involving human participants were reviewed and approved by Ethics committee of the medical faculty at the Otto-von-Guericke-University Magdeburg. Written informed consent to participate in this study was provided by the participants’ legal guardian/next of kin.

## Author Contributions

MK, M-PS and KK designed the experiment. MK collected data and wrote the manuscript. MK, M-KM and M-PS analyzed the data. KK and TZ supervised the study and provided research infrastructure. All authors reviewed the manuscript.

## Conflict of Interest

The authors declare that the research was conducted in the absence of any commercial or financial relationships that could be construed as a potential conflict of interest.
